# In Vitro Assessment of Single-Retainer Tooth-Colored Adhesively Fixed Partial Dentures for Posterior Teeth

**DOI:** 10.1155/2010/101095

**Published:** 2010-06-21

**Authors:** Tissiana Bortolotto, Carlo Monaco, Ioana Onisor, Ivo Krejci

**Affiliations:** ^1^Division of Cariology and Endodontics, School of Dentistry, University of Geneva, 19, Rue Barthélemy-Menn, CH-1205 Geneva, Switzerland; ^2^Department of Oral Sciences, Division of Prosthodontics and Maxillofacial Rehabilitation, University of Bologna, Via S. Vitale, 59, 40125 Bologna, Italy

## Abstract

The purpose of this paper was to investigate, by means of marginal adaptation and fracture strength, three different types of single retainer posterior fixed partial dentures (FPDs) for the replacement of a missing premolar. Two-unit cantilever FPDs were fabricated from composite resin, feldspathic porcelain, and fiber-reinforced composite resin. After luting procedures and margin polishing, all specimens were subjected to a Scanning Electron Microscopic marginal evaluation both prior to and after thermomechanical loading with a custom made chewing simulator comprising both thermal and mechanical loads. The results indicated that the highest score of marginal adaptation, that is, the closest score to 100% of continuous margins, at the *tooth-composite resin* interface was attained by the feldspathic porcelain group (88.1% median), followed by the fiber-reinforced composite resin group (78.9% median). The worse results were observed in the composite resin group (58.05% median). Fracture strength was higher in feldspathic porcelain (196N median) when compared to resin composite (114.9 N median). All the fixed prostheses made of fiber-reinforced composite resin detached from the abutment teeth before fracturing, suggesting that the adhesive surface's retainer should be increased.

## 1. Introduction

Two-unit cantilevered fixed partial dentures (FPDs) may be defined as retainers holding one or more unsupported free-end extensions. This type of prosthodontic rehabilitation has been used as an interim solution for restoring edentulous areas prior to and during implant therapy, instead of using a removable prosthesis [1]. While most of the studies available on cantilever prostheses refer mainly to the anterior area of the mouth [2–7], developments in the field of adhesion and minimally invasive therapy in terms of abutment preparation may also render this technique attractive for the replacement of a missing posterior tooth [8, 9]. 

Among the tooth-colored restorative materials available, fiber-reinforced composite resin is increasingly being used in prosthodontic rehabilitation [10–13]. The main advantages of this material are better stress distribution due to higher elasticity of the framework and relatively simplified laboratory procedures [14–18]. CAD/CAM technology allows for the construction of single unit restorations from industrially fabricated ceramic or composite resin blocks with predictable clinical success [19]. Similarly, the fabrication of CAD/CAM multiple unit restorations, that is, three-unit slot-inlay FPDs made of ceramic and composite-resin, is also possible [20]. However, failures with this type of design are frequently due to loss of retention from the abutments or to fractures within the ceramic or composite-resin material [21]. These failures occur either because the adhesive area provided by the abutment slot preparation is insufficient to withstand mastication forces, or because in three-unit FPDs both abutments are subjected to twisting forces [22] that can cause high stresses at the connector area and/or tooth-restoration interface; with the corresponding materials' fracture or debonding from one retainer. 

The major advantages of two-unit cantilever-inlay FPDs for single tooth replacement are that they involve less tissue damage, they are easier to clean, they are less explensive and that there is no chance of undetected debonding due to its single retainer [23]. In addition, twisting forces may be reduced, preventing the detachment of the restoration.

A high success rate of clinical longevity can be expected from two-unit cantilevered resin-bonded FPDs made of nickel chrome alloy [8]. Meanwhile, there is little information available on tooth-colored that is resin composite or ceramic, adhesively fixed two-unit cantilever prostheses for the replacement of missing posterior teeth. Understanding the biomechanics of 2-unit cantilevered resin-bonded FPDs made from tooth-colored materials is important in order to learn about the potential limitations of this restorative technique, but also to be able to select the most appropriate restorative material. Therefore, the aim of the present paper, was to evaluate the marginal adaptation and fracture strength of mesio-occlusal inlay-retained cantilever-FPDs made from feldspathic ceramic blocks, microfilled composite-resin blocks and a fiber-reinforced composite-resin. Because stress values at premolar cantilevers are lower than in molar cantilevered FPD [24], the ideal pontic dimensions should not exceed the mesio-distal dimension of a premolar. Thus, in the present paper a mesio-occlusal box preparation was used for framework support of a missing premolar. The null hypothesis tested was that marginal adaptation and fracture strength would fail to identify differences in the fatigue behavior of 2-unit cantilever FPDs made of resin composite, fiber-reinforced composite and feldspathic porcelain.

## 2. Materials and Methods

The materials used in the present study are listed in Table 1.

Caries-free human molars of nearly identical size and complete root growth were were procured from a private dental office with the understanding and oral consent of the patient. The teeth needed to be extracted due to periodontal reasons. They were stored in a 0.1% thymol solution for a maximum of 2 months after extraction. The teeth were randomly divided into three groups (*n* = 6). The apex of each root was sealed with an adhesive system (Syntac classic, Ivoclar Vivadent, Schaan, Liechtenstein) without removing the pulpal tissue. To simulate intratubular fluid flow, a cylindrical cavity was drilled 1.5 mm below the cementoenamel junction until the pulp chamber was reached. A metal tube with a diameter of 1.4 mm was luted into the cavity with the same adhesive system. Subsequently, the teeth were mounted on aluminium bases with micro hybrid composite-resin and the bases were immersed in an autopolymerizing acrylic resin (Technovit 4071; Heraeus-Kulzer, Friedrichsdorf, Germany) to an apical depth of two thirds of the root length to create a strong load-resistant support. Through a connecting silicone tube, the pulp chamber was evacuated with a vacuum pump (Vacubrand GmbH, & Co, Wertheim, Germany) and then filled with a bubble-free mixture of horse serum (PAA Laboratories GmbH, Linz, Austria) and phosphate-buffered saline solution (PBS; Oxoid Ltd, Basingstoke, Hampshire, England) with the aid of a 3-way valve, and finally connected to a serum infusion bottle. This bottle was placed vertically 34 cm above the specimen to simulate the normal hydrostatic pressure of 25 mm Hg within the tooth. 

**Figure 1:**
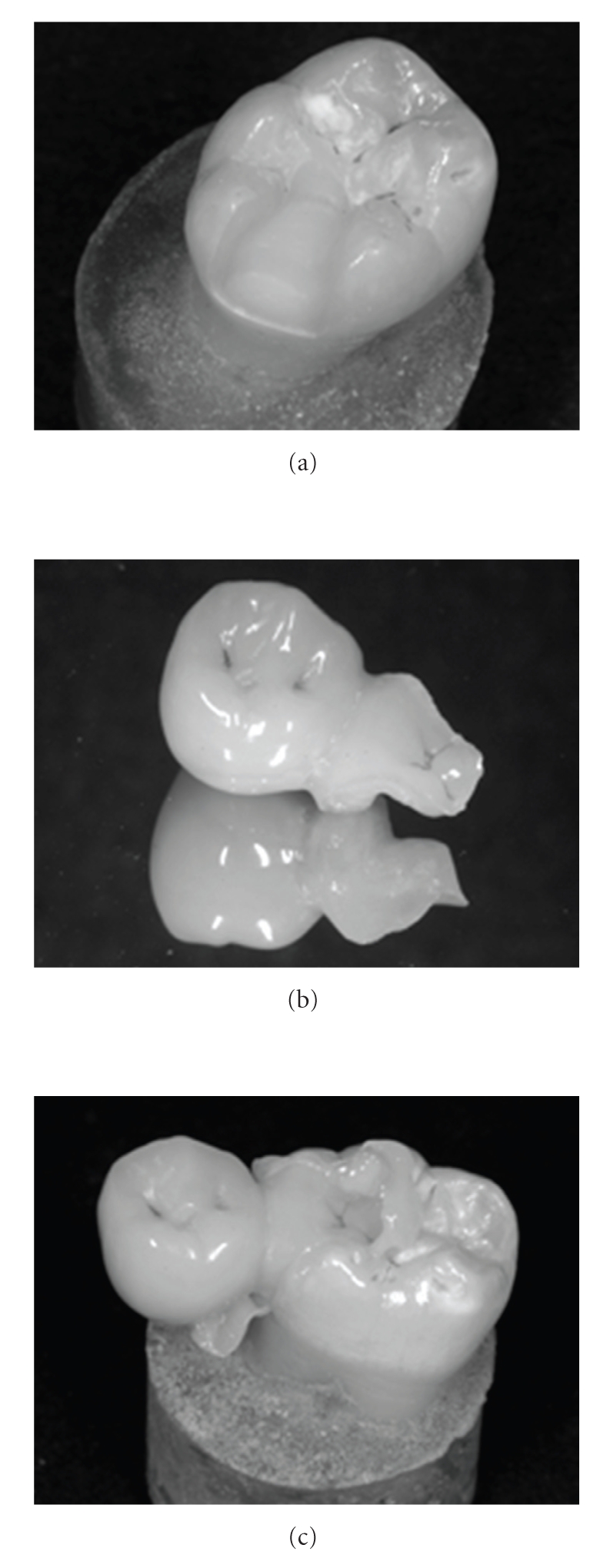
View of one cantilever FPD made of fiber reinforced composite. (a) A butt joint mesio-occlusal inlay cavity was prepared on the abutment tooth. (b) The cantilever bridge consisted on a premolar crown retained to the abutment by an inlay restoration. (c) The luting of the FPD was performed with a microhybrid restorative composite.

An inlay preapration (mesio-occlusal, butt joint margins on enamel) was made in each molar by the use of a rotating diamond instrument (80–25 *μ*m grain size, FG 8113NR, 3113NR, Intensiv SA, Viganello Switzerland) mounted on a red contra-angle handpiece (Sirius 180 XL, Micro-Mega, Besançon, France) under continuous water-cooling. The depth of the occlusal inlay was of 2 mm and the occlusal step was 4 mm. The interproximal step of 2 mm was used along with an axial depth of 1.6 mm and a faciolingual width of 3.5 mm (Figure 1(a)). 

The pontic measured 7 mm mesiodistally, which approximately corresponds to the size of a second premolar. The connectors of the inlay with the pontic (Figures 1(b) and 1(c)) were set to 3.5 × 3.5 mm, in agreement with a previous protocol [10]. After tooth preparation, the dentin surface was immediately sealed with a 3-step self-etching adhesive system (Syntac classic) [25]. Then, the adhesive system was removed from the enamel margins using a diamond instrument without touching the adhesively-sealed dentin [10]. 

For the construction of the fiber reinforced (FRC) cantilever FPDs, polyether impressions (Impregum Duo soft polyheter, 3M ESPE, Seefeld, Germany) were made using a simultaneous mixing technique following the manufacturer's instructions. Then, provisional restorations were made using Fermit N (IvoclarVivadent) without any cement and placed according to the clinical recommendations proposed by the manufacturer. The FRC system consists of two materials: glass fibers with different orientation (Vectris, IvoclarVivadent) and a microfilled composite (Adoro, IvoclarVivadent) for the veneering of the framework. The design of the fiberglass framework was first premodelled with a light polymerizing resin (Spectra Tray, Ivoclar Vivadent) to obtain the oval shape and its thickness checked on the molding model. This model was embedded in a transparent silicone impression paste to form a mold. The resin was removed, and the fibers were applied into the silicone-mould. The pre-impregnated “pontic” fibers were condensed in a deep-drawing polymerization process. After a cycle of vacuum-forming and after polymerizing by light in a special unit (VS1; IvoclarVivadent) for 10 minutes according to the manufacturer's recommendations, the FRC was airborne particle abraded (MicroEtcher CD, Danville Materials, San Ramon, CA, USA) using a small grain size of 27 mm at 2.5 bar of pressure for 10 seconds and treated with a silane coupling agent (Wetting agent; Ivoclar Vivadent). A sheet of wave fibers “frame” was placed upon the “pontic” structure and the cycle in the light curing unit (VS1) was repeated. The Adoro material was built incrementally and precured. The final polymerization/tempering were performed by means of light and heat (Lumamat 100; Ivoclar Vivadent). The additional tempering step at 104°C was done to maximize the strength and the surface quality of the restorations. 

For the construction of the feldspathic porcelain (FP) and composite-resin (CR) fixed prostheses an optical impression was made with the digital camera from the Cerec System (Sirona Dental Systems, Bensheim, Germany). The construction and milling of the prostheses was carried out using the Cerec 3 system (Software version 1.60 R980) according to a modified version of a protocol for the fabrication of three-unit Cerec prostheses. Feldspathic porcelain (Vitablocs Mark II; Vita Zahnfabrik, Bad Zächingen, Germany) and microhybrid composite (GC Corp, Tokyo, Japan) prefabricated blocks were the materials used for the construction of the prostheses. After the milling procedure, they were manually adjusted to the abutment tooth using coarse diamond instruments under continuous water cooling. 

In the case of the FRC prostheses, the provisional restorations made of Fermit were removed and the teeth's dentin surfaces (which were previously sealed with bonding) were airborne particle abraded (MicroEtcher CD, Danville Materials, San Ramon, CA, USA) for 2 seconds using aluminum oxide powder (grain size of 27 *μ*m) at a pressure of 2 bars. The intaglio surfaces of the FRC and CR abutments were also abraded following the precedent procedure but for 10 seconds. The intaglio surface of the ceramic group (FP) was etched with 5% hydrofluoric acid (Ceramics Etch, Vita Zahnfabrik, Germany) for 60 seconds followed by the application of a silanecoupling agent (Monobond S, IvoclarVivadent). The tooth surface, that is, enamel margin and airborne particle abraded adhesive-covered dentin, was treated using an adhesive system (Syntac Classic, IvoclarVivadent) after phosphoric acid selective enamel conditioning. A microhybrid light cured composite resin (Tetric Transparent, IvoclarVivadent) was used as the luting agent (Figure 1(c)). An ultrasonic technique was used for the seating of the restoration. After removing the excess resin, the luting composite resin was light activated with constant relative power density of 800 mW/cm^2^ (Optilux 501, Demetron/Kerr, Danbury, CT, USA,) for 60 s each from cervical, buccal, lingual, and occlusal surfaces. The margins of the restorations were then finished using 15 mm diamond instruments (Composhape, Intensiv, Lugano, Switzerland) and polished with flexible discs (Sof-Lex; 3M ESPE). 

After polishing of the margins (before loading) and after loading, the specimens were cleaned with rotating nylon brushes (Hawe Neos Dental, Bioggio, Switzerland) and toothpaste before making impressions for the replicas. One pair of replicas from both interproximal and occlusal boxes (Table 2) was procured from each cantilever prosthesis by using poly vinyl siloxane impressions (President Plus Light-body, Colténe AG, Altstätten, Switzerland). 

The impressions were then filled with epoxy resin (Epofix, Struers, Rodovre, Denmark) and gold sputtered (SCD 030, Provac, FL-9496 Balzers, Liechtenstein) for their observation in a Scanning Electron Microscope (XL20, Philips, NL-5600 Eindhoven, Netherlands). A quantitative evaluation of the marginal adaptation was performed at a 200x magnification by using a custom made module programmed within image processing software (Scion Image, Scion Corp, Frederik, MA, USA). Three margin segments that constituted the *total margin length* were analyzed on the SEM: approximal enamel, cervical enamel, and occlusal enamel (Table 2). The percentages of continuous margins were evaluated along the margin and separately for tooth-luting composite (TC) and luting composite-restoration (CI) interfaces. 

The specimens were mechanically loaded in a computer-controlled masticator with 1,200,000 cycles of 49N each, at a frequency of 1.7 Hz. A total of 3,000 thermal cycles of type 5°C to 50°C to 5°C were performed simultaneously. The chamber was automatically emptied after 2 minutes for 10 s with air pressure to avoid mixing the cold and warm water. The load cycles were transferred to the buccal cusp of the pontic. 

After the thermomechanical loading procedure, the fracture strength of each prosthesis was calculated by loading them to failure in a universal testing machine (Instron, Milan, Italy). The force was applied on the center of the pontics using a steel ball (5 mm diameter) at a crosshead speed of 1 mm/minute. To ensure a regular force distribution and minimize the transmission of local force peaks from the steel bar to the pontics' cusps, a layer of 0.5 mm thick tin foil was placed between both surfaces. The failure determination was set at a 10% loss of the maximum loading force. Radiologic examinations (Vistascan, Dürr Dental GmbH & Co. KG, Germany) were made to document the different fracture patterns. Types of failure due to the fracture strength test were described as “adhesive” (in the adhesive interface) and “cohesive” (within ceramic or composite material). Two main locations of the “adhesive” failures were distinguished: between luting composite and inlays' restorative material (fiber reinforced composite, feldspathic ceramic, or composite resin) and between tooth substrate and luting composite.

### 2.1. Statistical Analysis

The evaluation of the data was performed with Stata 9.0 for Windows. Shapiro-Wilk W test showed that the distribution of the data was not normal. Therefore, a Kruskal-Wallis test was used to detect whether there were differences in the median values of marginal adaptation at both TC and CI interfaces. Chi Square test was used to detect differences in fracture strength among groups feldspathic porcelain (VM) and composite resin (CR). Fiber reinforced composite group (FRC) was excluded from statistical analysis as will be explained in the results section. Multiple comparisons between groups were carried out with Bonferroni post hoc test.

## 3. Results

**Figure 2:**
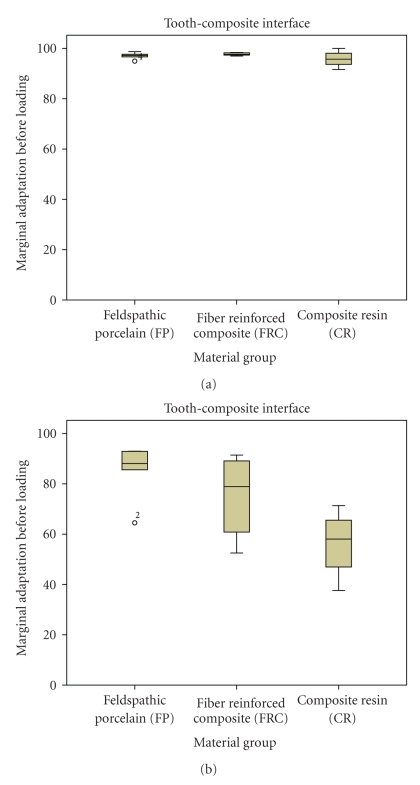
Marginal adaptation at the Tooth-Composite interface. Boxplots displaying the percentages of continuous margins of the three groups before (a) and after (b) thermal (3000x) and mechanical loading (1.2 million cycles). Median, 25% / 75% percentiles, and the highest and lowest not extremely values are shown.

**Figure 3:**
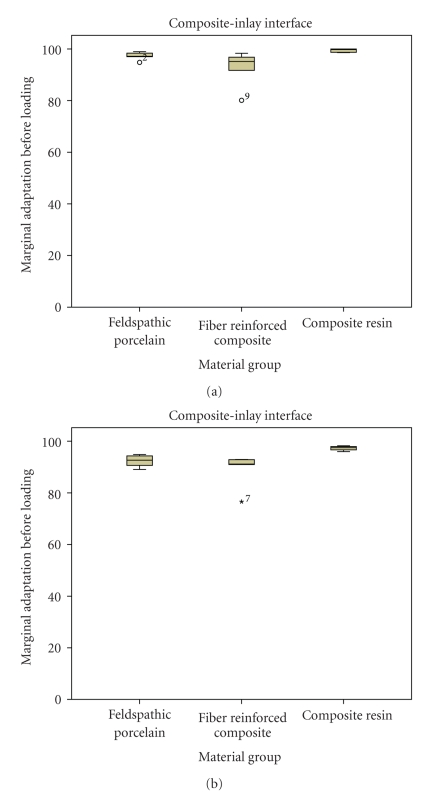
Marginal adaptation at the Composite-Inlay interface. Boxplots displaying the percentages of continuous margins of the three groups before (a) and after (b) thermal (3000x) and mechanical loading (1.2 million cycles). Median, 25%/75% percentiles, and the highest and lowest not extremely values are shown.

**Figure 4:**
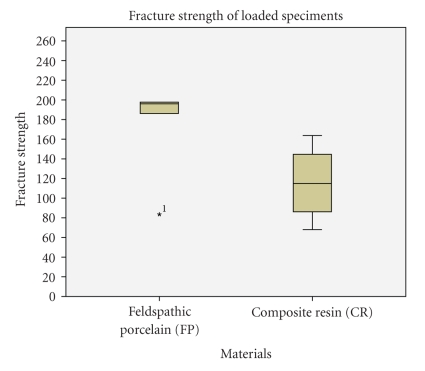
Results of fracture strength on loaded specimens. Note that FRC group has been excluded because the FPDs detached from the abutments before the fracture occured. Boxplots displaying the median (25%/75%) percentiles, and the highest and lowest not extremely values are shown Fracture strength in Newtons for FP: 196 (186.1/197.7) and for RC: 114.9 (86.2/144.6).

The results of marginal adaptation and fracture strength (expressed as the median, 25th and 75th quartiles) are detailed in Figures 2, 3, and 4. In respect to marginal adaptation at the *tooth-composite interface*, no significant differences were detected among FP, FRC, and RC prior to loading. The percentages of continuous margins were of 97.25, 97.65 and of 95.75, respectively. After loading, FP showed significantly better results (88.1% of continuous margins) when compared with CR (58.05% of continuous margins), as detailed in Figure 2. 

The percentages of continuous margins for FP, FRC, and RC at the *composite-inlay interface* were above 90%, both before (percentages of continuous margins of 97.15, 95.15 and 99.75, resp.) and after loading (92.65, 91.2 and 97.55, resp.) as can be observed in Figure 3. This indicated that the luting composite-inlay interface remained rather stable under fatigue conditions. 

When performing the fracture strength test on loaded specimens, all prostheses made of fiber reinforced composite (FRC) detached from the abutment before the fracture ocurred. Therefore, no fracture strength data could be procured from this group. A detailed observation of the inlays intaglio surface revealed that the luting composite remained attached to the inlay abutment and that detachments occurred principally between the luting composite and the tooth substrate. Regarding the other two materials, a higher fracture strength was reported for feldspathic porcelain (196N) in respect to Composite Resin (114.9N), as detailed in Figure 4. 

In respect to failure patterns, half of the prostheses made of feldspathic porcelain (FP) detached from the abutments. Adhesive failures were located between luting composite and tooth substrate and in some cases, remnants of enamel structure were still attached to the prosthesis. The other half failed due to cohesive fractures in the connector's area. All prostheses made of composite resin (CR) failed due to cohesive fractures within the restorative material, mainly located in the connectors' area.

## 4. Discussion

The results of the current study could reject the null hypothesis investigated. Marginal adaptation and fracture strength test could identify differences in the fatigue behavior of 2-unit cantilever FPDs made of resin composite, fiber-reinforced composite and feldspathic porcelain.

Two-unit resin-bonded cantilever bridges have been used for the replacement of a single missing anterior teeth [2–5]. Compared to conventional three-unit FPDs, easier cleaning, less biological damage, easier detection of debonding and decay underneath, as well as reduced twisting forces due to bonding to only one retainer have been reasons given for considering the clinical use of such a restorative technique [22]. Reasonably, the same arguments may promote the use of cantilever FPDs in the posterior area of the mouth.

In terms of the construction technique and materials' microstructure, the use of machinable composite-resin and/or feldspathic porcelain may be appealing for the construction of adhesive FPDs. The construction of milled FPDs from prefabricated blocks is not only faster, but material quality contributes to better long-term performance. A recent study demonstrated that in three-unit slot-retained FPDs fabricated from composite-resin and glass ceramic blocks, prosthesis fractures and debonding from the abutments led to a high percentage of failures [21]. To overcome such drawbacks, in this *in vitro* study, one-abutment inlay retained FPDs were evaluated for their fatigue resistance and fracture strength, to see if increasing the adhesive surface (inlays instead of slots) and limiting the number of retainers to one, could improve their mechanical performance. Feldspathic porcelain and microfilled composite-resin blocks were selected in the present study in agreement with a previous study that employed both materials for the production of CAD/CAM-generated slot-inlay FPDs [20]. The third material was FRC, which has been reported as being successfully used for the fabrication of three-unit posterior FPDs and also for the construction of cantilever bridges in the anterior region of the mouth [6, 7]. Three thousand thermal cycles together with 1.2 million cycles of occlusal loads were applied in a chewing simulator in order to fatigue the adhesive interfaces (Tooth-Composite and Composite-Inlay) and to assess if the three materials had a distinct influence on the stresses transferred to the abutment margins. Such stressing conditions are supposed to simulate a service time of 5 years [26]. In addition, fracture resistance after loading was calculated for each prosthesis to determine which material would better resist the impact of chewing forces in the posterior region. Finally, six FPDs were prepared on each group, following the methodology of recently published protocols in the field of inlay-retained adhesively fixed FPDs. To mention some examples, Ozcan et al. [27] evaluated the effect of different box preparations on the strength of glass fiber-reinforced composite inlay-retained fixed partial dentures; seven FPDs were tested per group. Keulemans et al. [28] evaluated the influence of retainer design on 2-unit cantilever FPDs; eight specimens were tested per group. Xie et al. [29] assessed the load-bearing capacity of fiber-reinforced composite FPDs with 4 framework designs; six specimens were evaluated per group. 

The high results, above 90% of continuous margins after loading, of marginal adaptation obtained at the *composite resin-inlay* interface showed that a high quality of bonding was achieved between prosthesis material and composite resin used as luting agent (Figure 2(b)). The ceramic surface was conditioned with hydrofluoric acid etching and further silanating. Both procedures ensured the formation of micromechanical retention and a proper wetting of the ceramic intaglio surface [30]. With respect to prostheses made out of composite resin, treatment of the internal surface with aluminium oxide airborne particle abrasion and silane has been shown to provide an efficient bonding to the luting composite resin material [10, 31, 32]. 

With respect to the *tooth-composite resin* interface, the results after loading showed that cantilevers made out of feldspathic ceramic (FP) demonstrated the highest marginal adaptation when compared with the other two groups (Figure 2(a)). Highest marginal adaptation means that the scores were close to 100% of continuous or close margins. The stiffer material delivered the highest quality of marginal adaptation [10]. As feldspathic porcelain has a higher modulus of elasticity than composite resin and rather similar to enamel (around 85 GPa), the more rigid material (FP) could have transferred less stresses to the margins in comparison to the composite resin group (CR), resulting in a more stable bond to the dental tissues when the FPDs were subjected to fatigue conditions. Regarding the marginal adaptation of the composite resin group (CR), the lowest percentages of continuous margins were obtained when compared to FP and FRC. These results were surprising, as for single unit restorations, a higher fatigue resistance has been observed when composite resin restorations were used instead of porcelain [33, 34]. This is due to the elastic behavior of resin composite during the loading cycle that can compensate for the forces that are transferred to the margins. However, in the case of cantilever FPDs, as the adhesive interface is subjected to higher stresses during the fatigue process, a more elastic material like composite resin can have an adverse effect on the marginal adaptation. This could serve as an explanation for the higher percentages of continuous margins observed in the FRC. As soon as resin composite was reinforced with fibers, no significant differences could be detected between the groups made of Felspathic Porcelain and Fiber Reinforced Composite. Resin composite reinforced with fibers helped to increase the stiffness of the FPD tested in this study, resulting in a similar stress transmission to the margins as with feldspathic porcelain.

During the fracture strength test materials fractures or detachments from the abutments occurred at a force of around 20 Kg (196N), which corresponds to a “light” chewing force in the clinical situation. A recent report stated that the mean values for the maximum bite force during mastication varied from 216 to 847N and that posterior fixed partial dentures should withstand loads of at least 500N [26]. In this *in vitro *study, only physiological chewing forces (49N = around 5 Kg) were used to load the FPDs. Under these experimental conditions, the highest results of fracture strength were attained by feldspathic porcelain (FP), as observed in Figure 3. Analysis of fractured specimens revealed that half of the specimens failed due to cohesive fractures in the connectors' area. The other half of the bridges failed in the adhesive interface. The examination of the detached FPDs revealed that the adhesive failure occurred between the luting composite and tooth substrate; in some specimens some remnants of enamel could be observed still attached to the ceramic surface after fracture. Said differently, cohesive failures occurred within enamel which means that the quality of the enamel-resin bond was not the weak link within the system. We speculate that the adhesive surface provided by the MO abutment preparation was insufficient as enamel, which is considered the most reliable substrate for adhesion, was limited to the margins of the conservative cavity preparation and adhesion relied mainly on dentin substrate. An increased adhesive area involving more enamel substrate could, in theory, improve the adhesive retention of this prosthesis design. Therefore, due to the presence of both cohesive and adhesive failures in the feldspathic porcelain group, further research should focus on the evaluation of all-ceramic cantilever FPDs with an increased adhesive area involving enamel and with a zirconia core to assess if with such a design, fracture resistance can be improved.

Fractures of FPDs made of resin composite occurred at a force of 114.9N (around 11 kg), which corresponds to a low mastication force. Such behaviour was expected due to the low fracture toughness of composite resin material and its poor ability to resist the propagation of cracks [15]. Therefore, composite resins without reinforcement, or at least with the current mechanical properties, should not be used for the fabrication of cantilever FPDs.

The fracture strength values of FPDs made of FRC could not be determined since they detached from the abutments prior to fracturing. Detachments occurred between the luting composite and the tooth substrate, suggesting that adhesion to the inlays intaglio surface was not the weak link. Problems related to the limited adhesive surface provided by the MO preparation may explain these detachments. A similar evaluation has been recently performed with three unit inlay-retained FPDs made of the same fiber reinforced material as the one used in this study; their fracture strength was 1373.4N after thermal mechanical stressing [11]. However, the retainers consisted of an MOD (mesio-occluso-distal) inlay on the premolar abutment and an MOLD (mesio-occluso-linguo-distal) onlay preparation on the molar. Considering that in the present study MO inlays were used as retainers, we speculate that debonding of the cantilever FPDs was influenced by the box dimensions and therefore, an insufficient surface area available for adhesion. Likewise, a recent study [28] evaluated the influence of different abutment preparations, that is, a proximal box, a step-box, a dual wing and a step-box-wing on the fracture strength of two-unit cantilever resin-bonded glass fiber reinforced composite FPDs. They concluded that a dual-wing retainer was the optimal design for replacing a single premolar by means of a two-unit cantilever FRC-FPD. Therefore, future research should evaluate the mechanical resistance of fiber-reinforced cantilever bridges with increased adhesive surfaces, for example an MOD inlay or a dual-wing retainer as the abutment.

## 5. Conclusion

Within the limitations of this *in vitro* study the following conclusions were drawn.

1. The null hypothesis was rejected as both, the evaluation of marginal adaptation after thermo mechanical loading and fracture strength testing were able to identify differences in the fatigue behavior of 2-unit cantilever FPDs made of resin composite, fiber-reinforced composite and feldspathic porcelain.
2. The marginal adaptation of feldspathic porcelain, the stiffest material, was comparable to the one of fiber-reinforced composite resin (FRC) (no significant differences between the materials). Composite resin (CR) FPDs produced the poorest marginal adaptation.
3. The highest fracture strength was attained by FPDs made of feldspathic porcelain and the lowest by FPDs made of composite resin. All composite resin FPDs fractured due to cohesive failures within the material, suggesting that the material was not sufficiently strong for this application. Fiber reinforced composite FPDs detached from the abutments before they fractured, suggesting that the adhesive surface was insufficient. In respect to feldspathic porcelain, both cohesive and adhesive failures at the luting composite—tooth interface were observed. Further evaluations with an increased abutment preparation and with a core reinforcement are necessary.

## Figures and Tables

**Table 1:** List of materials used in the present study.

Product (Group name)	Material type	Manufacturer	Batch number
Vitamark II (FP)	Feldspathic ceramic	Vita Zahnfabrik, Bad	2M1/6436
		Säckingen, Germany	
GN-1 (RC)	Microhybrid composite	GC Corporation, Tokyo, Japan	0000704 A
SR Adoro/Vectris (FRC)	Fiber reinforced	IvoclarVivadent, Schaan,	
	composite: glass fibers (Vectris) and a microfilled composite (Adoro)	Liechtenstein	

**Table 2:** Scheme of the quantitative margin analysis in the Scanning Electron Microscope. Two replicas were obtained from each cantilever FPD; one from the *mesial box* and the other one from the *occlusal box*. For the quantitative margin analysis, the enamel was divided into three segments: interproximal (segments a-b and c-d), cervical (segment b-c) and occlusal (segment a-d). All segments together constituted the *total margin length*.

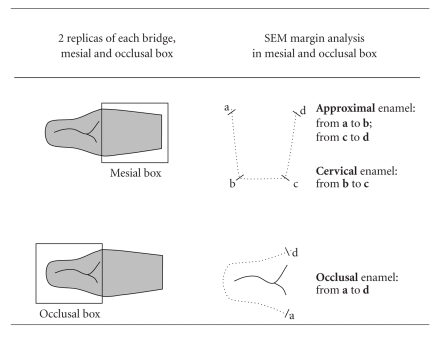

## References

[1] Pjetursson BE, Tan K, Lang NP, Brägger U, Egger M, Zwahlen M (2004). A systematic review of the survival and complication rates of fixed partial dentures (FPDs) after an observation period of at least 5 years IV. Cantilever or extension FPDs. *Clinical Oral Implants Research*.

[2] Kern M, Gläser R (1997). Cantilevered all-ceramic, resin-bonded fixed partial dentures: a new treatment modality. *Journal of Esthetic Dentistry*.

[3] Koutayas SO, Kern M, Ferraresso F, Strub JR (2002). Influence of framework design on fracture strength of mandibular anterior all-ceramic resin-bonded fixed partial dentures. *International Journal of Prosthodontics*.

[4] Kern M (2005). Clinical long-term survival of two-retainer and single-retainer all-ceramic resin-bonded fixed partial dentures. *Quintessence International*.

[5] Komine F, Tomic M (2005). A single-retainer zirconium dioxide ceramic resin-bonded fixed partial denture for single tooth replacement: a clinical report. *Journal of Oral Science*.

[6] Culy G, Tyas MJ (1998). Direct resin-bonded, fibre-reinforced anterior bridges: a clinical report. *Australian Dental Journal*.

[7] Auplish G, Darbar UR (2000). Immediate anterior tooth replacement using fibre-reinforced composite. *Dental Update*.

[8] Botelho MG, Leung KCM, Ng H, Chan K (2006). A retrospective clinical evaluation of two-unit cantilevered resin-bonded fixed partial dentures. *Journal of the American Dental Association*.

[9] Göehring TN, Peters OA, Lutz F (2001). Marginal adaptation of inlay-retained adhesive fixed partial dentures after mechanical and thermal stress: an in vitro study. *Journal of Prosthetic Dentistry*.

[10] Monaco C, Krejci I, Bortolotto T, Perakis N, Ferrari M, Scotti R (2006). Marginal adaptation of 1 fiber-reinforced composite and 2 all-ceramic inlay fixed partial denture systems. *International Journal of Prosthodontics*.

[11] Monaco C (December 2005). *Fiber reinforced composites: basic and clinical aspects of inlay fixed partial dentures*.

[12] van Heumen CCM, van Dijken JWV, Tanner J (2009). Five-year survival of 3-unit fiber-reinforced composite fixed partial dentures in the anterior area. *Dental Materials*.

[13] van Heumen CCM, Kreulen CM, Creugers NHJ (2009). Clinical studies of fiber-reinforced resin-bonded fixed partial dentures: a systematic review. *European Journal of Oral Sciences*.

[14] Krejci I, Boretti R, Giezendanner P, Lutz F (1998). Adhesive crowns and fixed partial dentures fabricated of ceromer/FRC: clinical and laboratory procedures. *Practical Periodontics and Aesthetic Dentistry*.

[15] Magne P, Perakis N, Belser UC, Krejci I (2002). Stress distribution of inlay-anchored adhesive fixed partial dentures: a finite element analysis of the influence of restorative materials and abutment preparation design. *Journal of Prosthetic Dentistry*.

[16] Monaco C, Ferrari M, Miceli GP, Scotti R (2003). Clinical evaluation of fiber-reinforced composite inlay FPDs. *International Journal of Prosthodontics*.

[17] Bouillaguet S, Schütt A, Marin I, Etechami L, Di Salvo G, Krejci I (2003). Replacement of missing teeth with fiber-reinforced composite FPDs: clinical protocol. *Practical Procedures &amp; Aesthetic Dentistry*.

[18] Vallittu PK (2004). Survival rates of resin-bonded, glass fiber-reinforced composite fixed partial dentures with a mean follow-up of 42 months: a pilot study. *Journal of Prosthetic Dentistry*.

[19] Reiss B (2006). Clinical results of cerec inlays in a dental practice over a period of 18 years. *International Journal of Computerized Dentistry*.

[20] Bindl A, Lüthy H, Mörmann WH (2003). Fracture load of CAD/CAM-generated slot-inlay FPDs. *International Journal of Prosthodontics*.

[21] Bortolotto T, Onisor I, Perakis N, Krejci I, Mörmann WH (2006). Composite and ceramic slot-retained adhesive bridges: effects of thermomechanical loading. *State of the Art of CAD/CAM Restorations- 20 Years of CEREC*.

[22] van Dalen A, Feilzer AJ, Kleverlaan CJ (2004). A literature review of two-unit cantilevered FPDs. *International Journal of Prosthodontics*.

[23] Wyatt CCL (2007). Resin-bonded fixed partial dentures: what’s new?. *Journal of the Canadian Dental Association*.

[24] Eraslan O, Sevimay M, Usumez A, Eskitascioglu G (2005). Effects of cantilever design and material on stress distribution in fixed partial dentures—a finite element analysis. *Journal of Oral Rehabilitation*.

[25] Stavridakis MM, Krejci I, Magne P (2005). Immediate dentin sealing of onlay preparations: thickness of pre-cured dentin bonding agent and effect of surface cleaning. *Operative Dentistry*.

[26] Wolfart S, Ludwig K, Uphaus A, Kern M (2007). Fracture strength of all-ceramic posterior inlay-retained fixed partial dentures. *Dental Materials*.

[27] Ozcan M, Breuklander MH, Vallittu PK (2005). The effect of box preparation on the strength of glass fiber-reinforced composite inlay-retained fixed partial dentures. *Journal of Prosthetic Dentistry*.

[28] Keulemans F, De Jager N, Kleverlaan CJ, Feilzer AJ (2008). Influence of retainer design on two-unit cantilever resin-bonded glass fiber reinforced composite fixed dental prostheses: an in vitro and finite element analysis study. *Journal of Adhesive Dentistry*.

[29] Xie Q, Lassila LVJ, Vallittu PK (2007). Comparison of load-bearing capacity of direct resin-bonded fiber-reinforced composite FPDs with four framework designs. *Journal of Dentistry*.

[30] Blatz MB, Sadan A, Kern M (2003). Resin-ceramic bonding: a review of the literature. *Journal of Prosthetic Dentistry*.

[31] Rocca GT, Krejci I (2007). Bonded indirect restorations for posterior teeth: from cavity preparation to provisionalization. *Quintessence International*.

[32] Rocca GT, Krejci I (2007). Bonded indirect restorations for posterior teeth: the luting appointment. *Quintessence International*.

[33] Brunton PA, Cattell P, Burke FJ, Wilson NH (1999). Fracture resistance of teeth restored with onlays of three contemporary tooth-colored resin-bonded restorative materials. *The Journal of Prosthetic Dentistry*.

[34] Magne P, Knezevic A (2009). Simulated fatigue resistance of composite resin versus porcelain CAD/CAM overlay restorations on endodontically treated molars. *Quintessence International*.

